# The first complete chloroplast genome sequences of *Ulmus* species by *de novo* sequencing: Genome comparative and taxonomic position analysis

**DOI:** 10.1371/journal.pone.0171264

**Published:** 2017-02-03

**Authors:** Li-Hui Zuo, Ai-Qin Shang, Shuang Zhang, Xiao-Yue Yu, Ya-Chao Ren, Min-Sheng Yang, Jin-Mao Wang

**Affiliations:** 1 Institute of Forest Biotechnology, Forestry College, Agricultural University of Hebei, Baoding, PR China; 2 Hebei Key Laboratory for Tree Genetic Resources and Forest Protection, Baoding, PR China; 3 Horticulture College, Agricultural University of Hebei, Baoding, PR China; Chinese Academy of Medical Sciences and Peking Union Medical College, CHINA

## Abstract

Elm (*Ulmus*) has a long history of use as a high-quality heavy hardwood famous for its resistance to drought, cold, and salt. It grows in temperate, warm temperate, and subtropical regions. This is the first report of Ulmaceae chloroplast genomes by *de novo* sequencing. The *Ulmus* chloroplast genomes exhibited a typical quadripartite structure with two single-copy regions (long single copy [LSC] and short single copy [SSC] sections) separated by a pair of inverted repeats (IRs). The lengths of the chloroplast genomes from five *Ulmus* ranged from 158,953 to 159,453 bp, with the largest observed in *Ulmus davidiana* and the smallest in *Ulmus laciniata*. The genomes contained 137–145 protein-coding genes, of which *Ulmus davidiana* var. *japonica* and *U*. *davidiana* had the most and *U*. *pumila* had the fewest. The five *Ulmus* species exhibited different evolutionary routes, as some genes had been lost. In total, 18 genes contained introns, 13 of which (*trnL*-TAA^+^, *trnL*-TAA^−^, *rpoC1*^-^, *rpl*2^-^, *ndhA*^-^, *ycf1*, *rps*12^-^, *rps*12^+^, *trnA*-TGC^+^, *trnA*-TGC^-^, *trnV*-TAC^-^, *trnI*-GAT^+^, and *trnI*-GAT) were shared among all five species. The intron of *ycf1* was the longest (5,675bp) while that of *trnF*-AAA was the smallest (53bp). All *Ulmus* species except *U*. *davidiana* exhibited the same degree of amplification in the IR region. To determine the phylogenetic positions of the *Ulmus* species, we performed phylogenetic analyses using common protein-coding genes in chloroplast sequences of 42 other species published in NCBI. The cluster results showed the closest plants to Ulmaceae were Moraceae and Cannabaceae, followed by Rosaceae. Ulmaceae and Moraceae both belonged to Urticales, and the chloroplast genome clustering results were consistent with their traditional taxonomy. The results strongly supported the position of Ulmaceae as a member of the order Urticales. In addition, we found a potential error in the traditional taxonomies of *U*. *davidiana* and *U*. *davidiana* var. *japonica*, which should be confirmed with a further analysis of their nuclear genomes. This study is the first report on *Ulmus* chloroplast genomes, which has significance for understanding photosynthesis, evolution, and chloroplast transgenic engineering.

## Introduction

Elm (*Ulmus*) has a wide distribution, from the tropics to cold temperate zones, and is mainly located in the former Soviet Central Asia, Kazakhstan, Siberia, Mongolia, Transbaikal, North Korea, and other countries in the Northern Hemisphere. There are 40 species worldwide, of which 25 are found in China, mainly to the north of the Yangtze River. *Ulmus* has resistance to drought, cold, and saline-alkali conditions, allowing it to grow in dry, barren dunes and chestnut soils [1–2]. Elm leaves are high in protein and can be used as animal feed and feed processing. Moreover, its fruit and bark can be used as medicine. Since *Ulmus* species have lush foliage, they are commonly grown in gardens.

The chloroplast is a semi-autonomous organelle in plant cells, and is one of three major genetic systems, the other two being the mitochondrion and the nucleus [3–5]. Chloroplasts are generally green, flat, and oval or spherical, and exist widely in the cytoplasmic matrix. Besides its role in photosynthesis, the chloroplast is closely associated with starch, fatty acid, pigment, and amino acid synthesis [6]. The chloroplast genome contains a large amount of genetic information. Due to its self-replication mechanism and relatively independent evolution, genetic information from the chloroplast is used to explore the occurrence, development, evolution of species and to develop the fields of plant genomics and bioinformatics [7]. In 1940, the first electron microscope image of chloroplasts was published by German scientists [8]. Then, Bedbrook and Bogorad [9] established the first chloroplast fingerprint of corn. Research on the chloroplast genome has gradually continued with the development of molecular biology and genomics. Scholars have found that, although the overall structure of the chloroplast genome is relatively conservative, it contains many mutations and some small structural changes among species, such as inversions, translocations, and insertion loss [10–12]. By now, over 700 plants have been sequenced, which has greatly improved chloroplast genome research. However, despite being one of the earliest trees to have been identified in the early third century, there is little genetic and genomic research on *Ulmus*. As a large plant branch, Ulmaceae comprises 230 elm species; however, there are no relevant reports on the elm chloroplast genome, which has hindered our understanding and progress of Ulmaceae evolution, species identification, genetic engineering, and other related research. Whatmore, there are still a lot of controversy about the taxonomic position of the Ulmaceae. In this report, we present the first complete chloroplast genomes of *Ulmus* by *de novo* sequencing. Using comparative genomics, we analyzed the characteristics of the chloroplast genomes of five *Ulmus* species and determined their phylogenetic positions.

## Materials and methods

### Test material

We collected test materials from the Black Dragon Mountain Nature Sanctuary in Zhangjiakou, PR China (charged by Zhangjiakou Forestry Bureau, the field studies did not involve endangered or protected species). We selected five native, wild species: *Ulmus pumila* L., *Ulmus laciniata* Mayr., *Ulmus davidiana* Planch., *Ulmus davidiana* Planch. var. *japonica* (Rehd.) Nakai, and *Ulmus macrocarpa* Hance. The leaves were cleaned, frozen in dry ice, and stored at -80°C refrigerator for subsequent analysis.

### Chloroplast genome sequencing

We used a high-salt, low-pH method to extract chloroplast genomic DNA from the leaves [13]. First, leaf homogenate was filtered and purified to obtain chloroplasts. We examined the samples under a microscope to assess chloroplast integrity, and then used DNase to digest nuclear, mitochondrial, and other DNA to prevent interference. After adding lysis solution, the chloroplasts were fully cleaved to release chloroplast DNA (cpDNA), and the chloroplast genome DNA was obtained by centrifugation, extraction, and purification. After passing an inspection, we sent the cpDNA to a company (Beijing ORI-GENE science and technology co., LTD) for high-throughput sequencing.

### *De novo* assembly and gap fillingand genome annotation

After filtering the raw data and removing of the impact of data quality (Phred score Cutoff-30), we obtained high-quality data. First, we used SOAPdenovo 2.01 (http://soap.genomics.org.cn/soapdenovo.html) [14] to perform the initial assembly and obtain the contig sequences. Then, we used BLAT 36 [15] (http://www.jurgott.org/linkage/LinkagePC.html) assembly to locate the long sequence of the near-edge species (*Morus notabilis*—KP939360.1, *Morus mongolica*- KM491711.2) of the chloroplast reference genome and obtain the relative positions of the contig sequences. According to the relative position of the contigs, we performed splicing and corrected assembly error. Finally, the whole framework maps of the chloroplast genomes were obtained.

We used the software GapCloser 1.12 (Gapcloser is part of software SOAPdenovo) (https://sourceforge.net/projects/soapdenovo2/files/GapCloser) to fill the gaps in the frame sequence diagram using high-quality short sequences, and then used generation sequencing to complement and confirm the remaining gaps and suspicious areas. Finally, we verified the long single copy section (LSC), short single copy section (SSC), and inverted repeat (IR) regional connectivity to obtain the ring-shaped complete chloroplast genome sequence. The chloroplast genome sequences were annotated with CpGAVAS [16] software (http://www.herbalgenomics.org/0506/cpgavas/analyzer/home) and DOGMA software, and then manually corrected.

### Selection pressure, co-linear and phylogenetic analysis

To analyze the environmental pressure in the process of the evolution of different elms, KaKs_Calculator 2.0 [17] (https://sourceforge.net/projects/kakscalculator2) were used to calculate Ka, Ks value of genes that with SNP differences. The codon preference were analyzed and maped by R software. We conducted a co-linear analysis of the *Ulmus* chloroplast genomes with published chloroplast genomes of other plants, including tobacco (*Nicotiana tabacum* NC_007500.1), *Arabidopsis thaliana* (NC_000932), poplar (*Populus* NC_009143) and mulberry (Moraceae NC_025772) species by GSV [18] (http://cas-bioinfo.cas.unt.edu/gsv/homepage.php). Firstly, the sequences of all chloroplast sequences were pair-wise compared by BLAST (http://www.jurgott.org/linkage/LinkagePC.html). Then, screen comparison fragments that similarity were over than 80% and the matching length longer than 100 bp for drawing by GSV. To determine the phylogenetic positions of *Ulmus* species, we selected other 42 species published in NCBI and used the common chloroplast protein-coding genes to explore the evolution of the chloroplast genomes of *Ulmus* species and to verify their taxonomic status by MEGA 6.0 [19]. CGView Server (http://stothard.afns.ualberta.ca/cgview_server/index.html) were used to analyze the genetic variation of the chloroplast genome of five *Ulmus* species.

## Results and analysis

### Basic characteristics of the elm chloroplast

**Fig 1:**
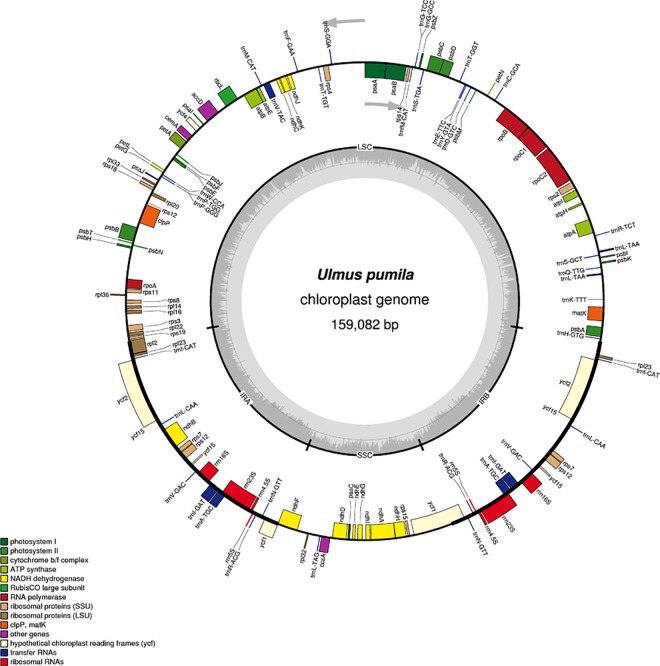
Gene map of the *U*. *pumila* chloroplast genome. Genes drawn inside the circle are transcribed clockwise, while genes outside are transcribed counterclockwise. Gene functional groups are color-coded.

Similar to other higher plants, the chloroplast structures of the five *Ulmus* species (S1–S4 Figs) had a typical quadripartite structure consisting of two single-copy regions (LSC and SSC) separated by a pair of IRs (Fig 1) [20–21]. The lengths of the chloroplast genomes ranged from 158,953 to 159,453 bp (Table 1), of which *U*. *davidiana* had the largest and *U*. *laciniata* had the smallest. The guanine-cytosine (GC) contents of the five species were similar, around 35.57%. Meanwhile, the length of the LSC exhibited greater variation, ranging from 87,633 to 88,547 bp, of which *U*. *macrocarpa* had the longest, followed by *U*. *davidiana* var. *japonica*, *U*. *pumila*, *U*. *laciniata*, and *U*. *davidiana*. Compared with the LSC, the SSC exhibited smaller variation, ranging from 18,835 to 18,868 bp, the longest was *U*. *davidiana* var. *japonica* and the smallest was *U*. *davidiana*. However, the difference in length of the IR followed the opposite trend as the SSC, the longest was *U*. *davidiana* (26,492 bp) and the shortest was *U*. *davidiana* var. *japonica* (26,017 bp). The number of protein-coding genes ranged from 137 to 145, of which *U*. *davidiana* var. *japonica* had the most and *U*. *pumila* had the fewest. These results indicate that several genes have been lost during evolution and different *Ulmus* species followed different evolutionary routes due to natural selection.

**Table 1:** Statistics on the basic features of the chloroplast genomes of five *Ulmus* species.

	***Ulmus pumila***	***Ulmus davidiana* Planch. var.**	***Ulmus macrocarpa***	***Ulmus davidiana***	***Ulmus laciniata***
Length (bp)	159082	159411	159439	159453	158953
GC content (%)	35.57	35.57	35.56	35.55	35.57
AT content (%)	64.43	64.43	64.44	64.45	64.43
LSC length (bp)	88103	88508	88547	87633	88032
SSC length (bp)	18850	18868	18853	18835	18846
IR length (bp)	26064	26017	26019	26492	26037
Gene number	137	145	142	143	139
Gene number in IR regions	37	39	39	40	37
Protein-coding gene number	83	89	88	88	85
Protein-coding gene (%)	60.58	61.38	61.97	61.54	61.15
rRNA gene number	8	8	8	8	8
rRNA (%)	5.84	5.52	5.63	5.59	5.76
tRNA gene number	46	48	46	47	46
tRNA (%)	33.58	33.1	32.39	32.87	33.09

**Fig 2:**
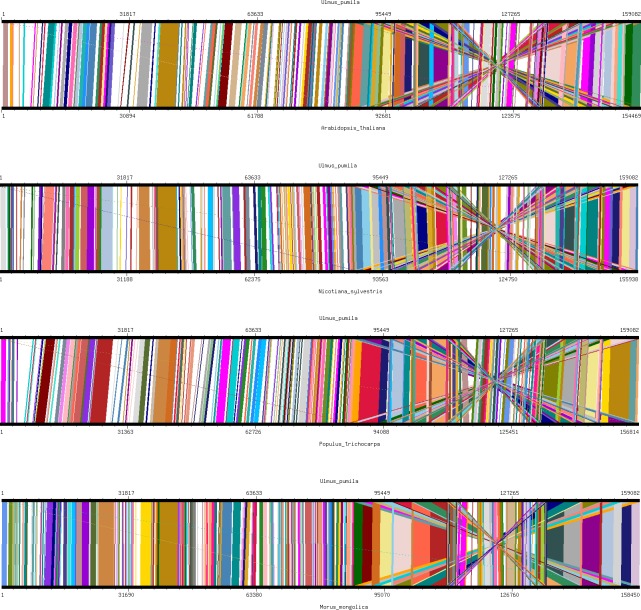
Co-linear analysis of various plant chloroplast genomes.

We used the co-linear method to analyze the chloroplast genomes of the five *Ulmus* species with their close relative Moraceae and other model plants (*N*. *tabacum*, *A*. *thaliana*, and *Populus*). The gene order of the chloroplast genomes of the five *Ulmus* species were highly conserved compared with that of other plants (Fig 2 and S5 Fig). Ulmaceae and Moraceae had the highest chloroplast genome homologies, while there were more common linear relationships among the other model plants. This demonstrates that the chloroplast genome has a high homology among various plants.

**Fig 3:**
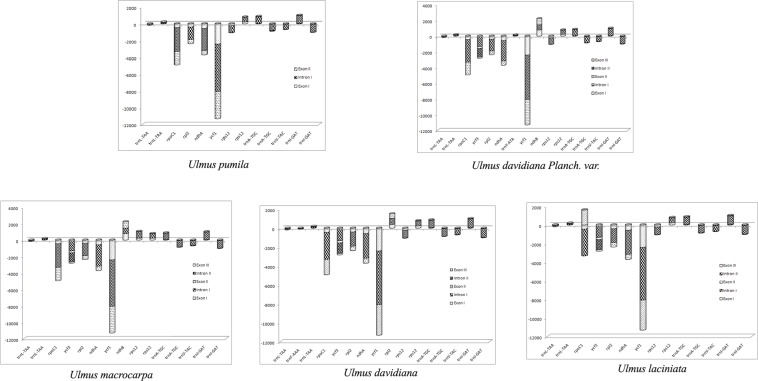
Intron length in the chloroplast genomes of five *Ulmus* species.

The results of the *Ulmus* chloroplast coding genes showed that they were similar to those of other plants, and most had no introns. In total, 18 genes contained introns (Table 2). Each *Ulmus* species had 13–18 of these genes: *U*. *davidiana* had 18 genes containing introns, followed by *U*. *davidiana* Planch. var. *japonica* (16), *U*. *macrocarpa* (16), *U*. *laciniata* (14) and *U*. *pumila* (13). All *Ulmus* species had 13 of the same intron-containing genes (*trnL*-TAA^+^, *trnL*-TAA^−^, *rpoC1*^−^, *rpl*2^−^, *ndhA*^−^, *ycf1*^−^, *rps*12^−^, *rps*12^+^, *trnA*-TGC^+^, *trnA*-TGC^−^, *trnV*-TAC^−^, *trnI*-GAT^+^, and *trnI*-GAT^−^), as well as the additional unique genes *trnY*-ATA and *ndhB* (*U*. *davidiana* var. *japonica*), *ndhB* (*U*. *macrocarpa*), and *trnF*-AAA and *rpl2* (*U*. *davidiana*). All the above genes contained one intron, except *ycf3*, which had two introns. The intron of *ycf1* was the longest (5,675 bp) while that of *trnF*-AAA was the smallest (53 bp) (Fig 3).

**Table 2:** Statistics of gene introns in the chloroplast genome of five *Ulmus* species.

**Gene**	**Strand**	*Ulmus* ***pumila***	***Ulmus davidiana* Planch. var.**	***Ulmus macrocarpa***	***Ulmus davidiana***	***Ulmus laciniata***
trnL-TAA	-	√	√	√	√	√
trnL-TAA	+	√	√	√	√	√
trnF-AAA	-	×	×	×	√	×
rpoC1	-	√	√	√	√	√
ycf3	-	×	√	√	√	√
rpl2	-	√	√	√	√	√
ndhA	-	√	√	√	√	√
trnY-ATA	+	×	√	×	×	×
ycf1	-	√	√	√	√	√
ndhB	+	×	√	√	×	×
rpl2	-	×	×	×	√	×
rps12	-	√	√	√	√	√
rps12	+	√	√	√	√	√
trnA-TGC	+	√	√	√	√	√
trnA-TGC	-	√	√	√	√	√
trnV-TAC	-	√	√	√	√	√
trnI-GAT	+	√	√	√	√	√
trnI-GAT	-	√	√	√	√	√
Total		13	16	16	17	14

### Chloroplast gene gain-loss events

Although the structure and composition of the chloroplast genomes of higher plants is relatively conserved, some species have structural variations and gene loss or metastasis due to evolution. In this study, we compared the five *Ulmus* species with proximal Moraceae, Cannabaceae species and the model plants *N*. *tabacum*, *A*. *thaliana*, and *Populus* (Table 3 and Table 4), we determined that *infA*, *lhbA*, *sprA*, and *ycf68* were readily lost during evolution, followed by *psbL*, *rps16*, *rrn5S*, *petB*, *petD*, *rrn16S*, *rrn23S*, *rrn4*.*5S*. The comparative results of five *Ulmus* species showed that *U*. *davidiana* had lost *trnH* compared with the other four elms. Moreover, *U*. *laciniata* had lost *ndhC* gene compared with the other four kinds of elms.

**Table 3:** Genes from the chloroplast genomes of various plant species.

Name of species	atpF	infA	lhbA	ndhC	petB	petD	psbL	psbZ	rpl32	rps12	rps16	rrn16S	rrn23S	rrn4.5S	rrn5S	sprA	trnA	trnC	trnD	trnE
**Ulmus davidiana**	+	-	-	+	-	-	-	+	+	+	-	+	+	+	+	-	+	+	+	+
**Arabidopsis thaliana**	+	-	-	+	+	+	+	+	+	+	+	+	+	+	-	-	+	+	+	+
**Ulmus pumila**	-	-	-	+	-	-	-	+	+	+	-	+	+	+	+	-	+	+	+	+
**Populus trichocarpa**	+	+	-	+	+	+	+	+	-	+	-	-	-	-	-	-	+	-	-	-
**Ulmus laciniata**	+	-	-	-	-	-	-	+	+	+	-	+	+	+	+	-	+	+	+	+
**Morus mongolica**	+	-	-	+	+	+	+	+	+	+	+	-	-	-	-	-	+	+	+	+
**Cannabis sativa cultivar Carmagnola**	+	-	+	+	+	+	+	-	+	+	+	-	-	-	-	-	+	+	+	+
**Ficus racemosa**	+	-	-	+	+	+	-	+	+	-	+	+	+	+	+	-	-	+	+	+
**Morus notabilis**	+	-	-	+	+	+	+	+	+	+	+	-	-	-	-	-	+	+	+	+
**Morus indica**	+	-	-	+	+	+	+	+	+	+	+	+	+	+	+	-	+	+	+	+
**Ulmus davidiana Planch. var. japonica (Rehd.) Nakai**	+	-	-	+	-	-	-	+	+	+	-	+	+	+	+	-	+	+	+	+
**Ulmus macrocarpa**	+	-	-	+	-	-	-	+	+	+	-	+	+	+	+	-	+	+	+	+
**Nicotiana sylvestris**	+	-	-	+	+	+	+	+	+	+	+	+	+	+	+	+	+	+	+	+
**Cannabis sativa cultivar Yoruba Nigeria**	+	-	-	+	+	+	+	+	+	+	+	-	-	-	-	-	+	+	+	+
**Total number of missing gene**	1	13	13	1	5	5	6	1	1	1	6	5	5	5	6	13	1	1	1	1

**Table 4:** Genes from the chloroplast genomes of various plant species.

Name of species	trnF	trnG	trnH	trnI	trnK	trnL	trnM	trnN	trnP	trnQ	trnR	trnS	trnT	trnV	trnW	trnY	ycf1	ycf15	ycf3	ycf68
**Ulmus davidiana**	+	+	-	+	+	+	+	+	+	+	+	+	+	+	+	+	+	+	+	-
**Arabidopsis thaliana**	+	+	+	+	+	+	+	+	+	+	+	+	+	+	+	+	+	-	+	-
**Ulmus pumila**	+	+	+	+	+	+	+	+	+	+	+	+	+	+	+	+	+	+	-	-
**Populus trichocarpa**	-	-	-	+	+	-	-	-	-	-	-	-	-	-	-	-	-	-	+	-
**Ulmus laciniata**	+	+	+	+	+	+	+	+	+	+	+	+	+	+	+	+	+	+	+	-
**Morus mongolica**	+	+	+	+	+	+	+	+	+	+	+	+	+	+	+	+	+	-	+	-
**Cannabis sativa cultivar Carmagnola**	+	+	+	+	+	+	+	+	+	+	+	+	+	+	+	+	+	+	+	+
**Ficus racemosa**	+	+	-	-	-	+	+	+	+	+	+	+	+	+	+	+	+	+	+	-
**Morus notabilis**	+	+	+	+	+	+	+	+	+	+	+	+	+	+	+	+	+	-	+	-
**Morus indica**	+	+	+	+	+	+	+	+	+	+	+	+	+	+	+	+	+	-	+	-
**Ulmus davidiana Planch. var. japonica (Rehd.) Nakai**	+	+	+	+	+	+	+	+	+	+	+	+	+	+	+	+	+	+	+	-
**Ulmus macrocarpa**	+	+	+	+	+	+	+	+	+	+	+	+	+	+	+	+	+	+	+	-
**Nicotiana sylvestris**	+	+	+	+	+	+	+	+	+	+	+	+	+	+	+	+	+	-	+	-
**Cannabis sativa cultivar Yoruba Nigeria**	+	+	+	+	+	+	+	+	+	+	+	+	+	+	+	+	+	-	+	-
**Total number of missing gene**	1	1	3	1	1	1	1	1	1	1	1	1	1	1	1	1	1	7	1	13

This gene loss phenomenon had also occurred in other plants. For example, the *ndh* gene family has been lost in the *Pinus* chloroplast genome [22]. Some of the genes *ycfL*, *ycf2*, *RPL23*, *infA*, and *rpsl6* have been lost in some angiosperms, and even all have been lost in some legumes [23]. The gene loss phenomenon may caused by the following two points: one side, different plants were subjected of different environmental pressures. The genes beneficial to plant growth were preserved, while some unimportant genes were lost in the long-term evolution. For example, most genes related to photosynthesis have been lost in parasitic plants (e.g., *E*. *virginiana*, *O*. *gracilis*, and *P*. *purpurea*). Another important reason may be due to gene transfer. A number of studies have reported that the gene migration phenomenon were occurred between the chloroplast, mitochondria and nuclear genomes, and this genes transfer has been continuing. For example, there are a large number of sequences from the chloroplast and its ancestral *Cyanobacteria* were found in the nuclear genome of arabidopsis, rice and tobacco [24–27].

### Codon preference analysis

**Fig 4:**
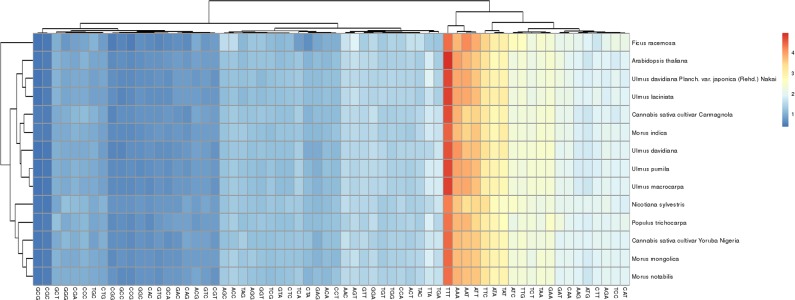
Codon distribution of all merged protein-coding genes. Color key: Red indicates a higher frequency and blue indicates a lower frequency.

In the *Ulmus* chloroplast genomes, 62% of the sequences were gene-coding regions, in which the vast majority of the sequences were protein-coding sequences. Based on the statistical analysis of all protein-coding genes and codons, we determined that all analytic varieties showed obvious codon preferences, and that the amino acids of proteins in the five *Ulmus* species were similar, of which TTT, AAT, AAA, ATT, and TTC had the highest frequencies (Fig 4). There was a high A/T preference in the third codon, which is very common in the chloroplast genomes of higher plants [28–32].

Codon degeneracy, whereby one amino acid has two or more codons, is very common. It is important biologically, as it can reduce the effects of harmful mutations in plants. If there were no selective pressure or mutation preference, nucleotide mutations in each amino acid site would be random, and the probability of synonymous codon usage would be the same. However, synonymous codon use is not random, and various genes in some species tend to use certain synonymous codons, which is known as synonymous codon usage bias. The relative synonymous codon usage (RSCU) is the ratio between the frequency of use and the expected frequency of a particular codon, and is an effective index to measure the degree of codon preference [33]. According to the RSCU (0.09–1.92) [34], synonymous codon preference can be partitioned into four models artificially: no preference (RSCU ≤ 1.0), low preference (1.0 < RSCU < 1.2), moderate preference (1.2 ≤ RSCU ≤ 1.3), and high preference (RSCU > 1.3).

**Fig 5:**
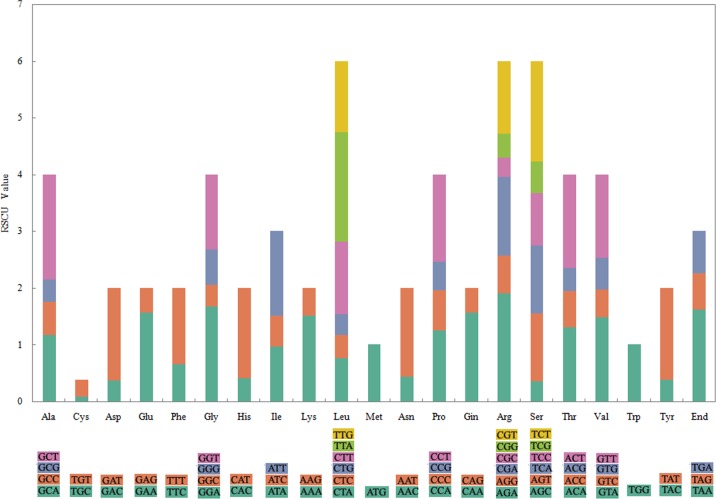
Codon content of 20 amino acid and stop codons in all protein-coding genes.

There were 64 codons encoding 20 amino acids in the chloroplast protein-coding genes of the five *Ulmus* species (Fig 5), and most of the amino acid codons, except methionine and tryptophan, had preferences; in total, we identified 31 codon preferences involving 18 amino acids and one stop codon. According to the synonymous codon preference partitions, 83.34% and 12.35% of all preferred codons exhibited high and moderate preferences, respectively. High codon preference is very common in chloroplast genes of higher plants, and is the main reason for the relative conservation of chloroplast genes.

### Selection pressure analysis of evolution

In genetics, Ka/Ks represents the ratio of non-synonymous substitution (Ka) to synonymous substitution (Ks). This ratio can determine whether selective pressure is acting on a particular protein-coding gene. In evolutionary analyses, it is important to understand the rate of synonymous and non-synonymous mutations. It is generally believed that mutations are not affected by natural selection, but that non-synonymous mutations are affected by natural selection. If Ka/Ks > 1, the gene is affected by positive selection, if Ka/Ks = 1, the gene is affected by neutral evolution, and if Ka/Ks < 1, the gene is affected by negative selection.

**Fig 6:**
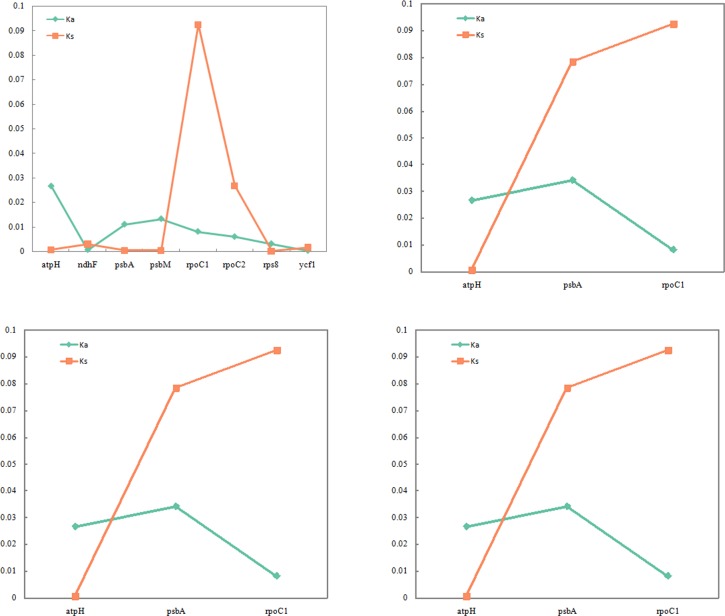
The Ka and Ks values of five *Ulmus* species. A–D represent the Ka and Ks values of *U*. *davidiana* var. *japonica*, *U*. *macrocarpa*, *U*. *davidiana*, and *U*. *laciniata* with respect to *U*. *pumila*.

**Fig 7:**
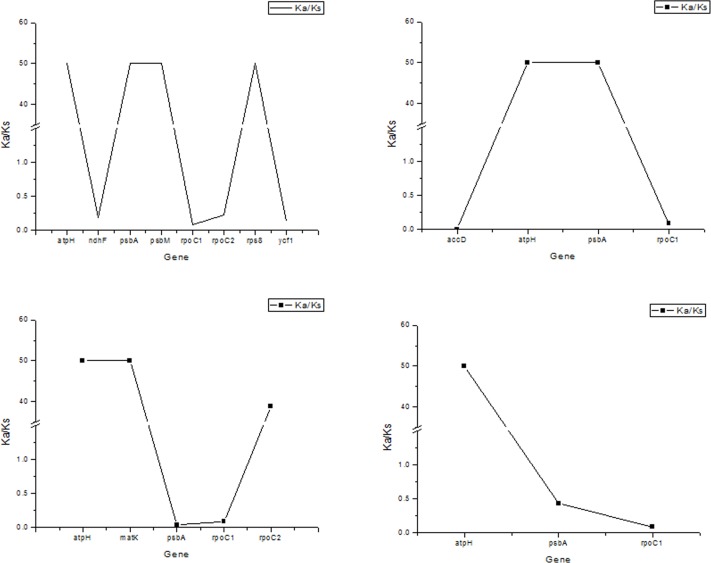
Ka/Ks values of five *Ulmus* species. A–D represent the Ka/Ks values of *U*. *davidiana* var. *japonica*, *U*. *macrocarpa*, *U*. *davidiana*, and *U*. *laciniata* with respect to *U*. *pumila*.

We found 8, 4, 5, and 3 protein-coding genes that exhibited single nucleotide polymorphisms, with the most observed in *U*. *davidiana* var. *japonica* and the fewest in *U*. *laciniata* (Fig 6). The Ka and Ks values of all *Ulmus* species were low (< 0.1), indicating that the *Ulmus* chloroplast protein-coding genes are relatively well conserved. Further analysis of Ka/Ks was used to determine whether selective pressure has acted on protein-coding genes. The genes *atpH*, *psbA*, *psbM*, and *rps8* in *U*. *davidiana* var. *japonica* were subjected positive selective pressure, whereas *ndhF*, *rpoC1*, *rpoC2*, and *ycf1* were subjected negative selection (Fig 7). Similarly, *atpH* and *psbA* were subjected to positive selective pressure, *accD* and *rpoC1* were subjected to negative selection in *U*. *macrocarpa*. Moreover, *atpH*, *matK*, and *ropC2* were subjected to positive selective pressure, psbA and *rpoC1* were subjected to negative selection in *U*. *davidiana*. Finally, *atpH* was subjected to positive selective pressure, *psbA* and *rpoC1* were subjected to negative selection in *U*. *laciniata*. All above indicated that the evolutionary route of chloroplast genes were different among different elms. For example, compared with *U*. *pumila*, *psbA* gene was subjected positive selective pressure in *U*. *davidiana* var. *japonica* and *U*. *macrocarpa*, whereas *psbA* gene was subjected negative selection in *U*. *davidiana* and *U*. *laciniata*. This indicates that the *Ulmus* chloroplast genomes have been influenced by different environmental pressures during evolution and this may the main reason for the difference of genes number in five *Ulmus* species.

### IR contraction analysis

**Fig 8:**
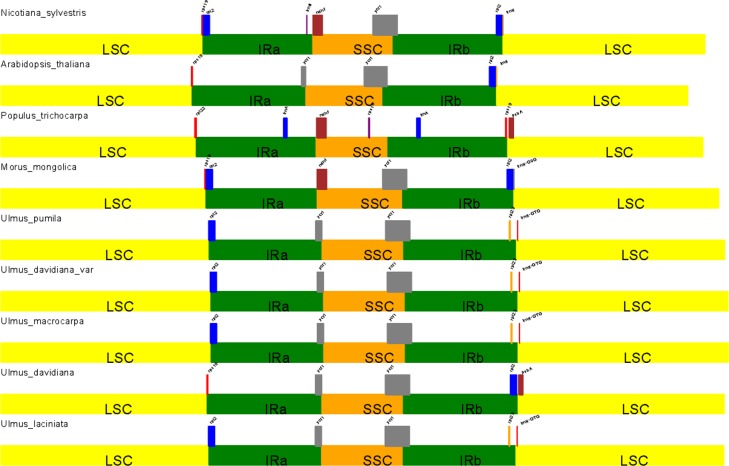
IR contraction analysis of various plant species.

Although the IR region of the chloroplast genome is considered to be the most conserved region, border region contraction and expansion is common during evolution and is the main driver of chloroplast genome length variability. In this study, we compared the IR-LSC and IR-SSC boundary conditions of the five *Ulmus* species with those of Moraceae, *N*. *tabacum*, *A*. *thaliana*, and *Populus* (Fig 8). The IRa/LSC boundaries of *U*. *pumila*, *U*. *laciniata*, *U*. *davidiana* var. *japonica*, *U*. *macrocarpa*, Moraceae, and *N*. *tabacum* all expanded and extended into *rpl2*. However, *rpl2* gene did not exist in *U*. *davidiana*, *N*. *tabacum*, *Populus*, and the IRa/LSC boundary expansion extended into *rps19* and *rps22*, respectively. The IRa/SSC regions of the five elms and *A*. *thaliana* all expanded into *ycf1*. The IRb/SSC boundaries of all plants extended into *ycf1*, although *Populus* had lost the *ycf1* gene. IRb/LSC boundaries of all *Ulmus* species extended into *rpl23*, with the *trnH* located downstream, except in *U*. *davidiana*. However, the IRb/LSC boundaries of *U*. *davidiana*, *N*. *tabacum*, *A*. *thaliana*, and Moraceae extended into *rpl12*.

### Phylogenetic analysis

**Fig 9:**
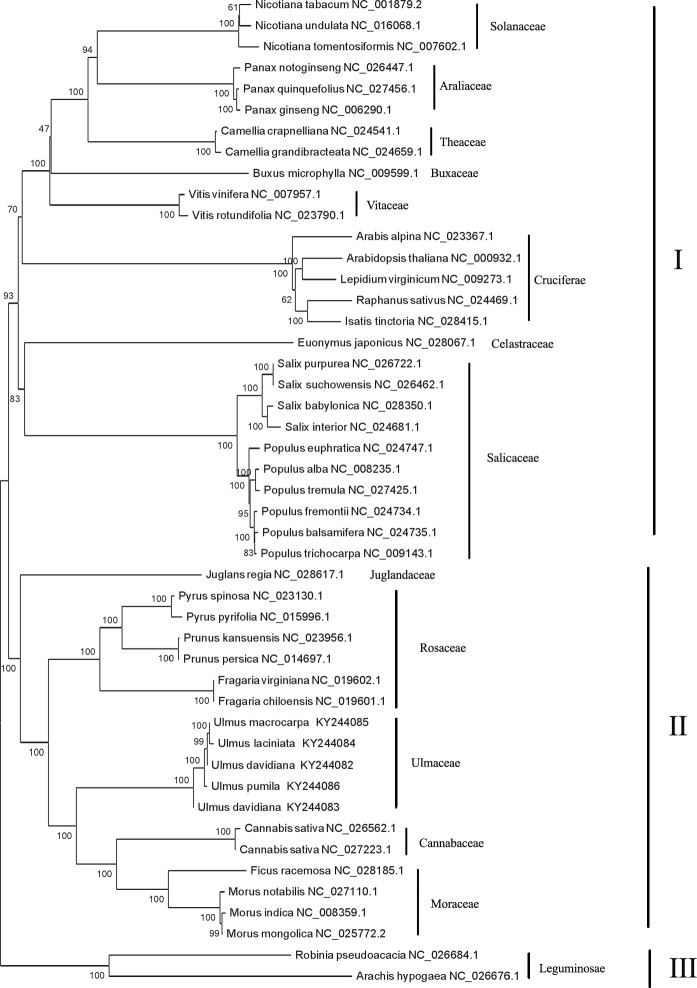
The ML phylogenetic tree of the Ulmaceae clade based on same protein-coding genes. Numbers above or below the nodes are bootstrap support values.

To determine the phylogenetic positions of *Ulmus* species, we performed a phylogenetic analysis using 52 common chloroplast protein-coding genes from 42 species published in NCBI. The clusters were well supported and the test scores of most of the branch nodes reached 100%, indicative of high reliability. From the analysis, all 47 plants were divided into three classes; the first class consisted of 27 species in Solanaceae, Araliaceae, Theaceae, Buxaceae, Vitaceae, Cruciferae Celastraceae, and Salicaceae, while the second class consisted of 18 species in Juglandaceae, Rosaceae, Ulmaceae, and Moraceae. The third class only consisted of two species that *Robinia pseudoacacia* and *Arachis hypogaea* (Fig 9).

The first class can also be subdivided into two categories. In the first category, the phylogenetic relationship of Salicaceae and Celastraceae were closer. Furthermore, Populus and Salix could be distinguished completely in Salicaceae. It is demonstrated that *P*. *alba* and *P*. *tremula* belong to white poplar and have the closest phylogenetic relationship inside the Populus, whereas *P*. *euphratica* have rather distant phylogenetic relationships that separated into an independent branch. In the second category, Solanaceae, Araliaceae, Theaceae, Buxaceae and Vitaceae have closer relationship, while Cruciferae have more distant relatives. The second class can also be subdivided into 3 categories and almost all of the test scores of the branch nodes reached 100%. Compared with other plants, *Juglans regia* had a further genetic relationship and were separated into an independent category. In the Rosaceae family (the second category), Pyrus (*P*. *spinosa*, *P*. *pyrifolia*) had closer relationship with Amygdalus (*P*. *persica*, *P*. *kansuensis*), while Strawberry (*F*. *virginiana*, *F*. *chiloensis*) had further relationship. The third category were consisted of Ulmaceae, Moraceae, and Cannabaceae, in addition, all plants belonged to Urticales. Therefore, the resulting phylogenetic topologies analysis were with high bootstrap supports and provided the evolutionary placement and relationship of Ulmaceae. The results strongly support the position of Ulmaceae as a member of the order Urticales. Finally, *Robinia pseudoacacia* and *Arachis hypogaea* (Leguminosae) had further relationship with other 45 plants and separated into an independent branch.

As the primitive group of Urticales, previous studies on the phylogeny of *Ulmus* mainly focused on its phenotypic traits (e.g., petals, pollen, epidermal micromorphology, embryology, anatomy, and pericarp) and chromosome, which lacked molecular evidence. However, due to the convergence of some characteristics and simplification of its flower structure, it is difficult to classify *Ulmus*. Ulmaceae classification has been a focus debate among taxonomists, including the position of Ulmaceae and the relationship between *Ulmus* species with other Urticales species. The clustering results showed that Moraceae and Cannabaceae were closest to Ulmaceae, followed by Rosaceae. Wiegrefe [35] obtained the same conclusion from an analysis of a chloroplast DNA restriction enzyme map of Ulmaceae. Ulmaceae, Cannabaceae, and Moraceae all belonged to Urticales, and the chloroplast genome clustering results were consistent with their traditional taxonomies. The results strongly support the position of Ulmaceae as a member of the order Urticales. However, some of the chloroplast genome classifications of the five *Ulmus* species were inconsistent with traditional taxonomy. In particular, traditional taxonomy places the phylogenetic relationship of *U*. *davidiana* and *U*. *davidiana* var. *japonica* closer together, and there may be other errors that need to be identified.

Traditional taxonomy is more dependent on plant phenotype to distinguish species; however, phenotypic traits, such as samara and leaf shape, are frequently influenced by the environment. Therefore, there are many homonyms and synonyms in classical taxonomy that are inconsistent with their origin. With a relatively independent evolutionary path, the chloroplast genome is widely used to analyze genetic evolution and identify plant species. However, the chloroplast genome is smaller and contains less genetic information. To determine whether there are errors in the traditional taxonomy of *U*. *davidiana* and *U*. *davidiana* var. *japonica*, further analyses using their nuclear genomes should be conducted.

**Fig 10:**
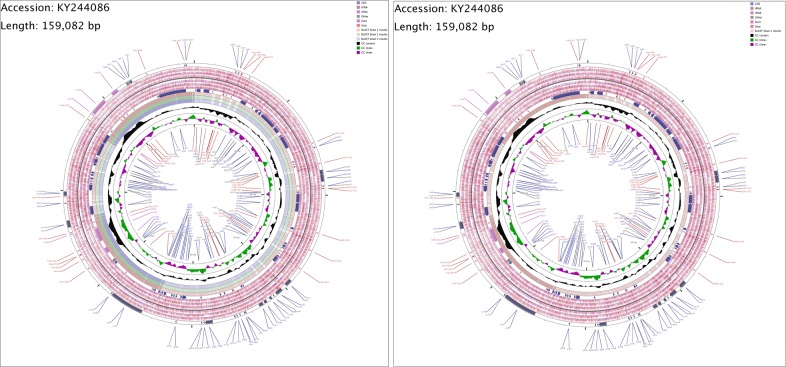
Comparison of chloroplast genome of five *Ulmus* species. Fig 10A and Fig 10B were take *U pumila* L as contrast, In Fig 10A blast 1 to blast 3 were *U*. *davidiana* Planch.var. *japonica* (Rehd.) Nakai, *U*. *macrocarpa* Hance., and *U*. *davidiana* Planch. Fig 10B blast 1 was *U*. *laciniata* Mayr. Ring 1: Forward strand features from *Ulmus pumila* L. Rings 2,3,4: Forward strand start and stop codons in reading frames 3,2,1. Rings 5,6,7: Reverse strand start and stop codons in reading frames 1,2,3. Ring 8: Forward strand features from *U*. *pumila* L. Rings 9,10,11: Blast result of chloroplast genome 1,2,3. Ring 12: GC content of *U*. *pumila* L.

To analyze the genetic variation of the *Ulmus* species, we used the chloroplast genome of *U*. *pumila* as a reference and analyzed single nucleotide variations (SNV) and indel variations in their chloroplast genomes using the GCView Server (http://stothard.afns.ualberta.ca/cgview_server/index.html) (Fig 10). *U*. *davidiana* var. *japonica* had the most mutation sites (total, 360; SNV, 286; indel, 74), followed by *U*. *davidiana* (total, 351; SNV, 296; indel, 55), *U*. *laciniata* (total, 318; SNV, 260; indel, 58), and *U*. *macrocarpa* (total, 297; SNV, 251; indel, 46) (S1 Table). Due to maternal inheritance of the chloroplast genome, most of the genes in the chloroplast were conserved, except for small portions of genes. Most of the mutations were located between genes, whereas eight genes in *U*. *davidiana* var. *japonica*, six in *U*. *davidiana* and *U*. *macrocarpa*, and seven in *U*. *laciniata* contained variations. The gene *rpoC1* had the most variation sites.

## Conclusions

In this study, we report five *Ulmus* species chloroplast genomes by *de novo* sequencing. The lengths of the chloroplast genomes from five *Ulmus* species ranged from 158,953 to 159,453 bp, with the largest in *U*. *davidiana* and the smallest in *U*. *laciniata*. The number of protein-coding genes ranged from 137 to 145, of which *U*. *davidiana* var. *japonica* and *U*. *pumila* had the most and *U*. *laciniata* had the fewest. Almost all protein-coding sequences and amino acid codons showed obvious codon preferences. Selection pressure analysis indicated that different *Ulmus* chloroplast genomes have been influenced by different environmental pressures during long-term evolution and this may the main reason for the difference of genes number in five *Ulmus* species. The phylogenetic analysis results strongly supported the position of Ulmaceae as a member of the order Urticales. However, we found a potential error in the traditional taxonomies of *U*. *davidiana* and *U*. *davidiana* var. *japonica*, which should be confirmed with a further analysis of their nuclear genomes. This study is the first report on the chloroplast genome of *Ulmus* and has significance for research on photosynthesis, evolution, and chloroplast transgenic engineering.

## Supporting information

S1 Fig Gene map of the *U*. *davidiana* Planch. var. *japonica* chloroplast genome. Genes drawn inside the circle are transcribed clockwise, while genes outside are transcribed counterclockwise. Gene functional groups are color-coded.
(TIF)

S2 Fig Gene map of the *U*. *macrocarpa* chloroplast genome. Genes drawn inside the circle are transcribed clockwise, while genes outside are transcribed counterclockwise. Gene functional groups are color-coded.
(TIF)

S3 Fig Gene map of the *U*. *davidiana* chloroplast genome. Genes drawn inside the circle are transcribed clockwise, while genes outside are transcribed counterclockwise. Gene functional groups are color-coded.
(TIF)

S4 Fig Gene map of the *U*. *laciniata* chloroplast genome. Genes drawn inside the circle are transcribed clockwise, while genes outside are transcribed counterclockwise. Gene functional groups are color-coded.
(TIF)

S5 Fig Co-linear analysis of various plant chloroplast genomes. (TIF)

S1 Table Chloroplast genome variation of five elm. (XLS)
