# Supplementary figures and images for: The first complete chloroplast genome sequences of Ulmus species by de novo sequencing: Genome comparative and taxonomic position analysis
Source: PLoS One. 2017 Feb 3;12(2):e0171264. doi: 10.1371/journal.pone.0171264 (PMC5291543; doi:10.1371/journal.pone.0171264)

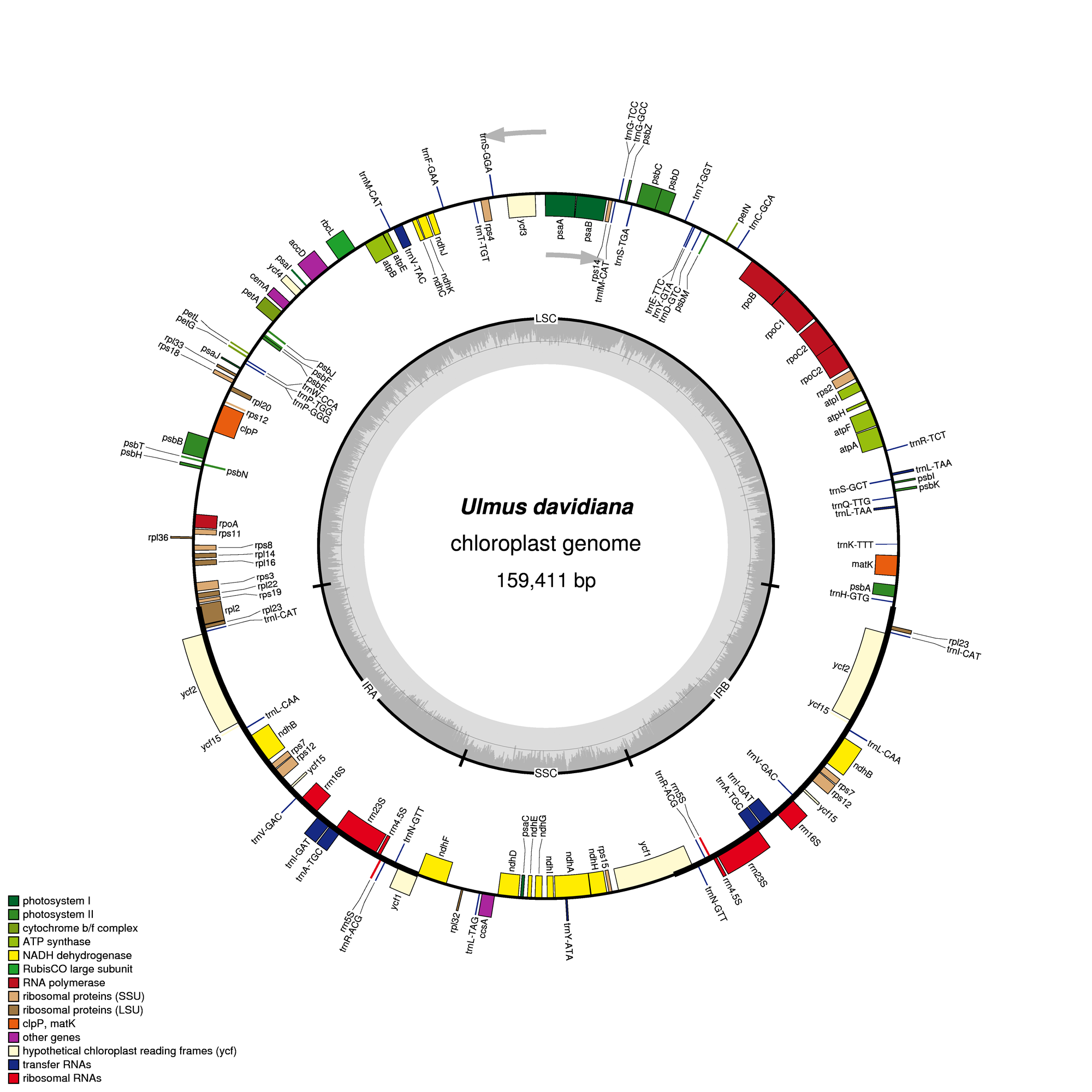

Supplement: S1 Fig — Genes drawn inside the circle are transcribed clockwise, while genes outside are transcribed counterclockwise. Gene functional groups are color-coded. (TIF) [file pone.0171264.s001.tif]

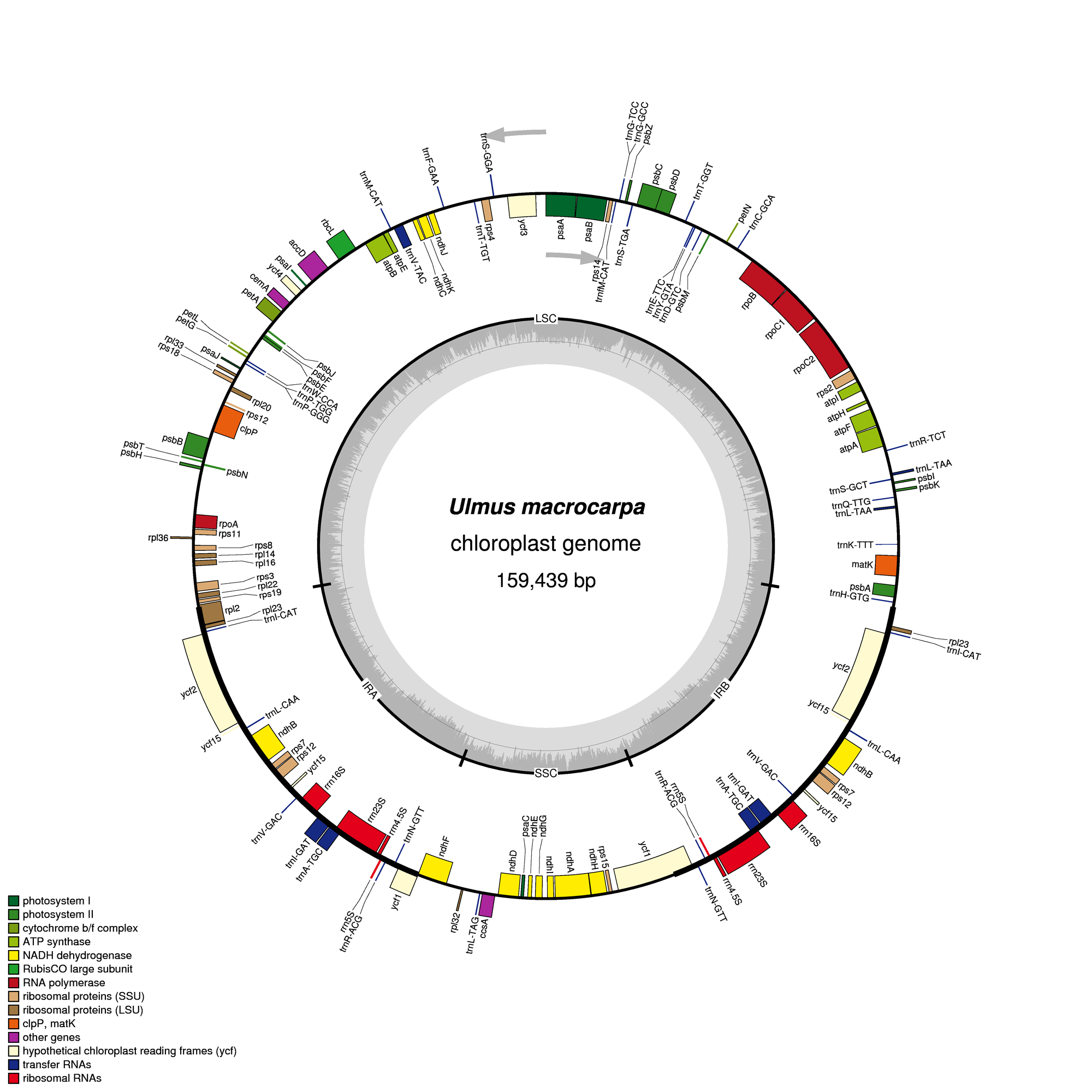

Supplement: S2 Fig — Genes drawn inside the circle are transcribed clockwise, while genes outside are transcribed counterclockwise. Gene functional groups are color-coded. (TIF) [file pone.0171264.s002.tif]

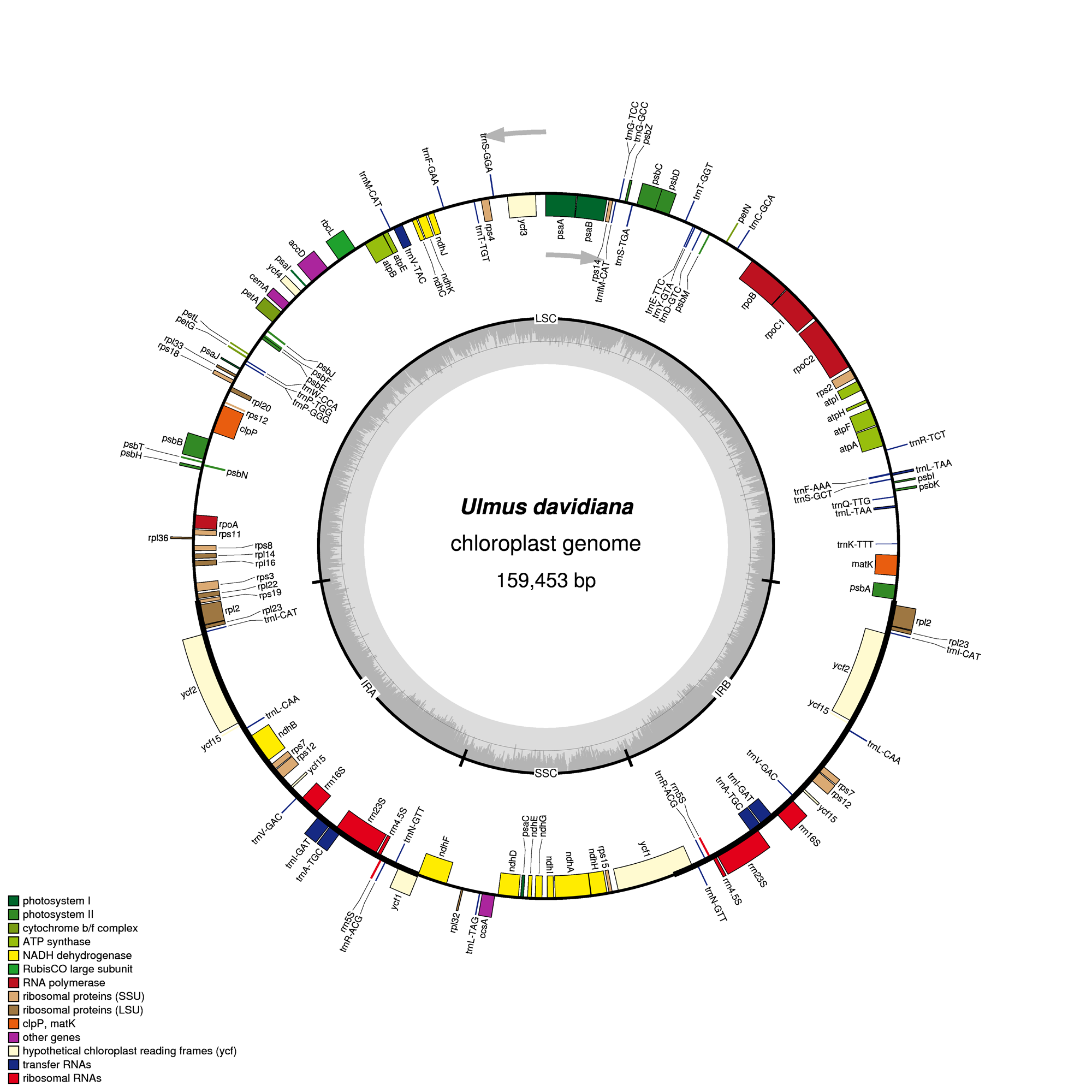

Supplement: S3 Fig — Genes drawn inside the circle are transcribed clockwise, while genes outside are transcribed counterclockwise. Gene functional groups are color-coded. (TIF) [file pone.0171264.s003.tif]

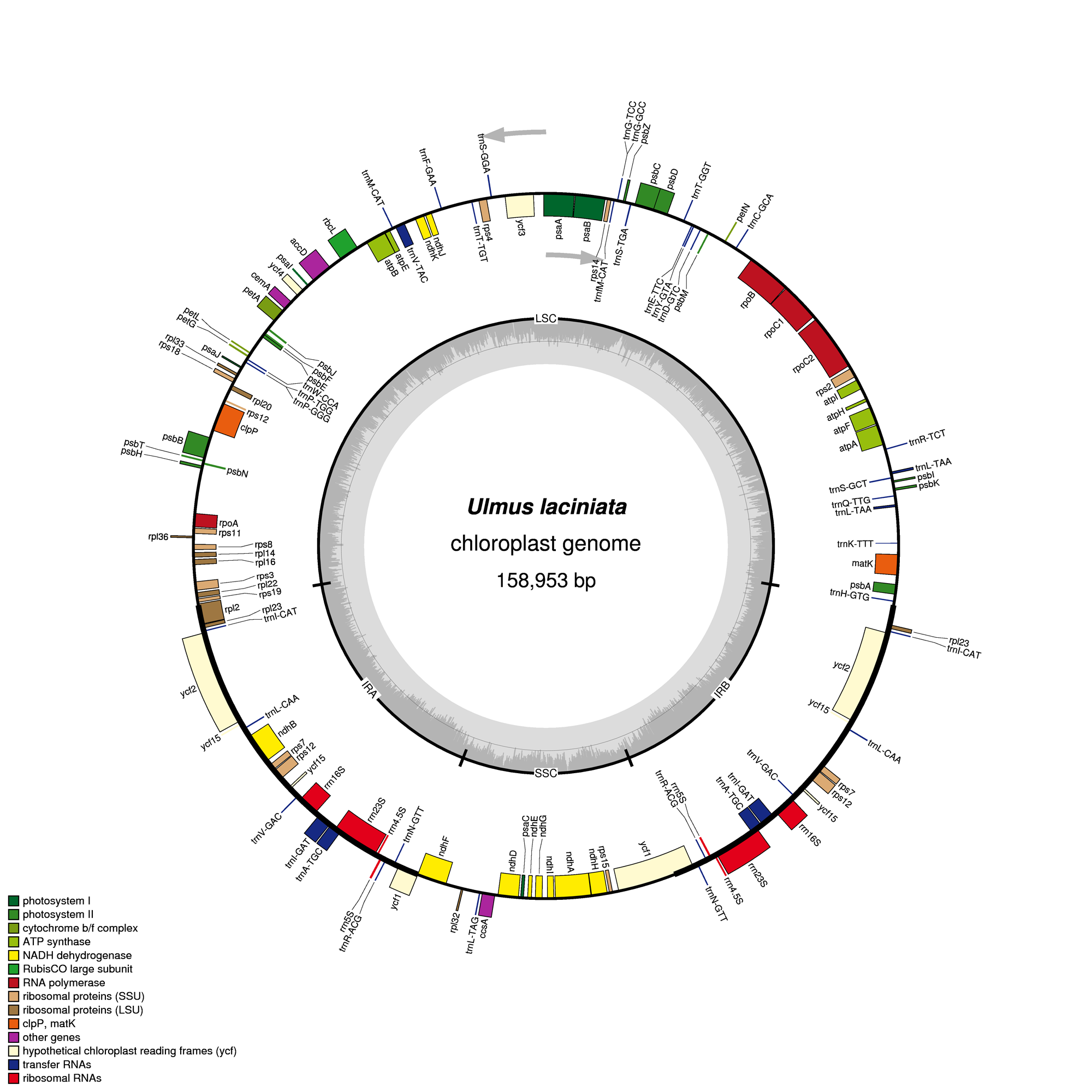

Supplement: S4 Fig — Genes drawn inside the circle are transcribed clockwise, while genes outside are transcribed counterclockwise. Gene functional groups are color-coded. (TIF) [file pone.0171264.s004.tif]

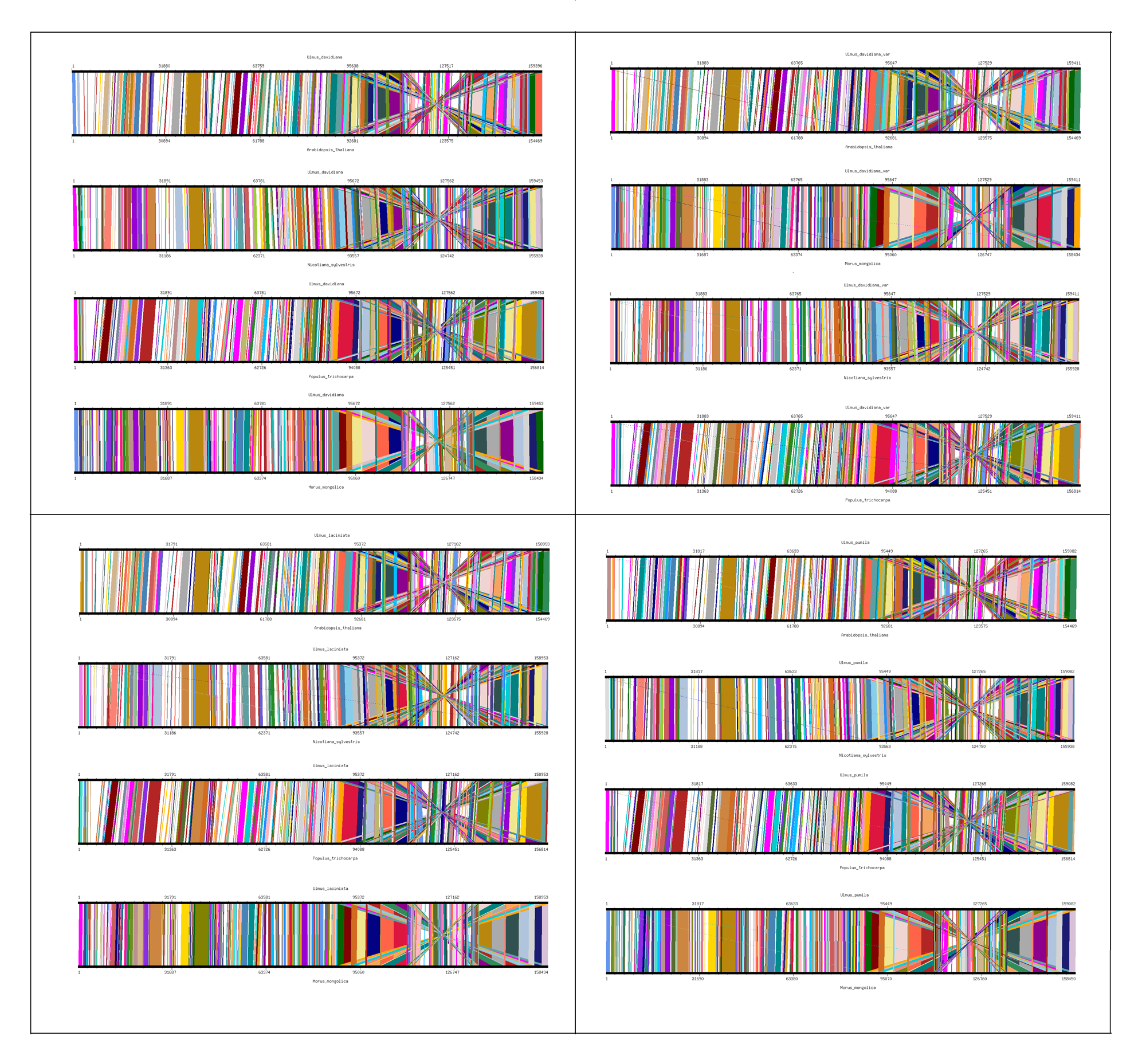

Supplement: S5 Fig — (TIF) [file pone.0171264.s005.tif]
